# Alginate-Based Beads Containing *Artemisia absinthium* L. Extract as Innovative Ingredients for Baked Products

**DOI:** 10.3390/gels12010043

**Published:** 2026-01-01

**Authors:** Alessandro Candiani, Giada Diana, Vincenzo Disca, Yassine Jaouhari, Margherita Stampini, Stefano Salamone, Federica Pollastro, Jessica Baima, Flavia Prodam, Sabrina Tini, Marta Bertolino, Lorella Giovannelli, Lorena Segale, Jean Daniel Coïsson, Marco Arlorio

**Affiliations:** 1 Department of Pharmaceutical Sciences, University of Piemonte Orientale, Largo Donegani 2, 28100 Novara, NO, Italy; alessandro.candiani@uniupo.it (A.C.); giada.diana@uniupo.it (G.D.); vincenzo.disca@uniupo.it (V.D.); yassine.jaouhari@uniupo.it (Y.J.); margherita.stampini@uniupo.it (M.S.); stafano.salamone@uniupo.it (S.S.); federica.pollastro@uniupo.it (F.P.); lorella.giovannelli@uniupo.it (L.G.); jeandaniel.coisson@uniupo.it (J.D.C.); marco.arlorio@uniupo.it (M.A.); 2 Department of Health Sciences, University of Piemonte Orientale, Via Solaroli 17, 28100 Novara, NO, Italy; jessica.baima@uniupo.it (J.B.); flavia.prodam@uniupo.it (F.P.); sabrina.tini@uniupo.it (S.T.); 3 Department of Agricultural, Forest and Food Sciences, University of Torino, Largo Paolo Braccini 2, 10095 Grugliasco, TO, Italy; marta.bertolino@unito.it

**Keywords:** microencapsulation, *Artemisia absinthium* L., ionotropic gelation, spray drying, alginate, enriched biscuits

## Abstract

*Artemisia absinthium* L. is a medicinal plant well known for the bitterness of its sesquiterpenoids. To mask its intense taste while preserving these active compounds, an ethanolic extract (AAE) was prepared, and two microencapsulation techniques (spray drying and ionotropic gelation) were investigated under different process conditions. The best-performing formulation was selected for larger-scale production and a characterisation of the microparticles (MPs) was carried out. MPs were then incorporated into baked products (biscuits), which were subsequently characterised for proximate composition, total phenolic content (TPC) and antioxidant activity (AA). Bitter compounds were quantified through HPLC-DAD. A panel test was conducted on 50 volunteers, which compiled a satisfactory questionnaire. Ionotropic gelation proved to be the most suitable technique for producing AAE alginate-based MPs for incorporation into biscuit dough, yielding a product with a desirable particle size and flowability. The biscuits still retained a significant amount of TPC and AA, indicating that microencapsulation is a suitable strategy. Data from the acceptance questionnaire revealed that biscuits containing MPs loaded with absinthin-rich extract were comparable to the control ones regarding overall acceptance. In conclusion, a promising product was developed that effectively masks the bitterness of appetite-modulating bioactive compounds, with significant health-promoting potential. However, further investigation into the biological effects (e.g., hormonal responses, feelings of hunger, etc.) of these baked products is required.

## 1. Introduction

Bitter compounds are widespread throughout nature and occur in a wide variety of plants, animals, and fungi [1,2,3]. Synthesized primarily as defence and deterrent agents, these molecules often exhibit a certain degree of toxicity [4]. Despite this, many of them have long been employed in traditional medicine and are commonly used to flavour foods and beverages [5,6].

Within this broad chemical landscape, sesquiterpenoid lactones, characteristic constituents of the Asteraceae family, stand out for their ability to interact with receptors of the hTAS2Rs family [7,8]. These G protein-coupled receptors mediate the perception of bitterness and are expressed not only on the tongue but also in numerous extra-oral tissues [9,10,11]. Notably, activation of hTAS2Rs in the gastrointestinal tract triggers physiological responses that extend well beyond taste perception, including delayed or inhibited gastric emptying and modulation of endocrine hormone release, ultimately contributing to enhanced satiety [12].

Among bitter botanical species, *Artemisia absinthium* L. (commonly known as wormwood) is particularly noteworthy. This perennial shrub from the Asteraceae family is well known both for its long-standing use in traditional medicine and as the key ingredient of the historic liqueur absinthe. Numerous pharmacological activities have been attributed to the plant, for example, antioxidant, free radical scavenging, anti-inflammatory, hypoglycaemic and hypocholesterolemic effects, as well as antibacterial, antiviral, and antifungal properties [13,14,15,16,17]. Additionally, it is widely utilized in the food and beverage industry for the production of bitters, aperitifs, and spirits [17]. These diverse bioactivities stem from its rich phytochemical profile, which comprises terpenoids, polyketides, phenylpropanoids, and other compounds [18]. Among them, absinthin, a dimeric sesquiterpene lactone, plays a central role in the plant’s characteristic intense bitterness and is known to activate the hTAS2R46 receptor [19]. Although ethanolic extracts of *Artemisia absinthium* represent a valuable source of this molecule, their use in oral formulations is limited by their extremely bitter taste.

To address this challenge, microencapsulation presents a promising strategy for masking bitterness while preserving the integrity and biological activity of the extract’s components. Two technologies are particularly suitable for this purpose: spray drying and ionotropic gelation. Spray drying converts a liquid formulation into a dried powder by atomizing it into a hot air stream, which rapidly evaporates the solvent and produces solid microparticles. Compared with other microencapsulation methods, spray drying offers several advantages, including shorter processing times, lower operating costs, high throughput due to continuous processing, ease of scale-up, applicability to both heat-sensitive and heat-stable materials, flexible capacity design, and precise control over particle size [20].

Conversely, ionotropic gelation relies on the ability of certain polyelectrolytes to crosslink in the presence of counterions, leading to the formation of microencapsulated systems [21]. This approach is advantageous because it does not require organic solvents or exposure to high temperatures, making it particularly suitable for formulations containing sensitive or thermolabile active compounds. It is a simple, rapid, and cost-effective method that operates under mild conditions [22].

In this context, the present study aims to compare two microencapsulation techniques (spray drying and ionotropic gelation) for the production of a dry ethanolic extract of *A. absinthium*, with the dual objective of masking its pronounced bitterness and preserving its molecular integrity, ultimately enabling targeted activation of gastrointestinal bitter receptors and the potential physiological benefits associated with their stimulation.

## 2. Results and Discussion

### 2.1. Preparation and Characterisation of Artemisia absinthium L. Extract

The ethanolic extraction and the subsequent evaporation of the solvent at reduced pressure led to the obtainment of 99 g (6.6%) of a dark green syrup.

*A. absinthium* extract was characterised for the main sesquiterpene lactones according to ^1^H NMR. Purification led to afford the bis-guaianolide absinthin (1.30%) and other similar molecules such as artabsin (0.48%), anabsin (0.21%) and anabsinthin (0.31%).

The TPC of the initial *A. absinthium* extract, evaluated through the Folin–Ciocâlteu test, resulted in a concentration of 3.10 ± 0.32 mg CE/mL, and the antioxidant activity of the initial absinthe ethanolic extract, assessed with the ABTS assay, turned out to be 1.16 ± 0.04 mg TE/mL.

### 2.2. Microparticles Obtained by Spray Drying

Preliminary studies to select a suitable excipient for spray drying led to the choice of ethylcellulose as the polymeric carrier, at concentrations of 5–6% in ethanol and 9–11% in acetone. The reason for the selection of these two solvents lies in the final intended use. Indeed, considering that the final product would be a baked product to be orally ingested, the solvent selected to solubilize the absinthe extract was acetone for formulations 1–4, while it was ethanol for formulations 5–8.

Spray drying proved to be an effective technique for obtaining products with satisfactory properties. All formulations were successfully processed, yielding light-green powders with recoveries ranging from 45% to 65%, which are consistent with the values commonly reported in the literature for laboratory-scale spray dryers [23,24]. These results may be attributable to the very small size of the MPs produced; the smallest particles may have been carried to the equipment filter along with the evaporated solvent and, consequently, lost.

All eight batches of MPs had a mean particle size below 35 µm regardless of the formulation. The small particle size, often a positive and required feature in the technological field, in this case, could not be a useful characteristic because of the possible difficulties encountered during the further handling of the systems and their not-so-easy workability.

The dynamic angles of repose of all eight powders ranged between 36 and 40°, indicating fair flowability, according to the European Pharmacopoeia [25]. These data demonstrated the adequacy of this product for the intended final use.

Even though fair flowability and the homogeneous narrow particle size distribution were achieved, the not-so-high nor satisfying recovery values, joined with the small mean diameter of the MPs, led to discarding this option for the further work phase.

### 2.3. Microparticles Obtained by Ionotropic Gelation

Preliminary studies led to the selection of Phospholipon^®^ 90G (Plp 90G) as an appropriate stabilizer for the formulations undergoing ionotropic gelation.

**Figure 1:**
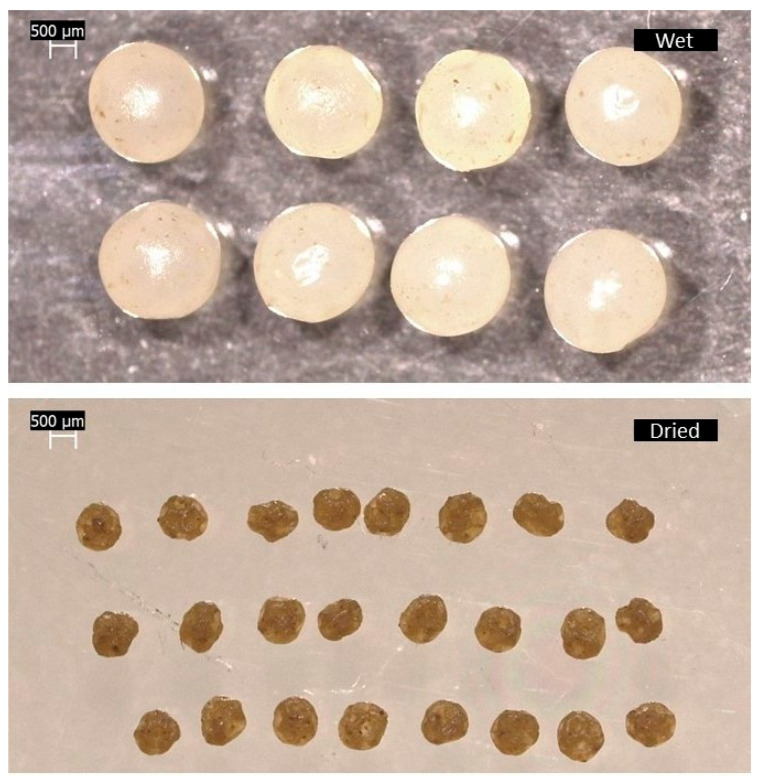
Wet (**top**) and dried (**bottom**) MPs obtained by ionotropic gelation.

Ionotropic gelation enabled the production of pale yellowish spherical beads containing very small, darker spots, corresponding to the absinthe extract, uniformly dispersed within the alginate matrix in their freshly produced state (wet). Wet MPs were bigger than the dried ones, as clearly observable from Figure 1, with a mean diameter of 2.336 mm, which decreased to 0.909 mm after the drying process. The sphericity of the wet beads was underlined by the shape factor (SF) value of 0.95, determined by ImageJ analysis (SF = 1 indicates perfect sphericity). Furthermore, wet MPs exhibited a more regular and smoother surface compared to the dried ones. Although the dried MPs largely retained their spherical shape, they showed some irregularities, as reflected by the shape factor (SF = 0.89), and acquired the same brown color as the original absinthe extract (Figure 1).

**Figure 2:**
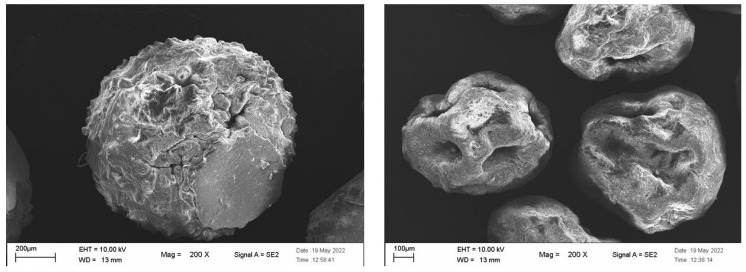
Static (**left**) and dynamic (**right**) dried MP SEM images.

SEM images revealed that the two drying methods were responsible for distinct morphologies in the final microparticles. Specifically, static drying resulted in particles with a uniform surface and a noticeable flattened area where the MPs had rested on the Petri dish. In contrast, dynamic drying generated particles with a rougher surface, characterized by visible depressions, although no fractures or holes were observed in their structure (Figure 2).

**Figure 3:**
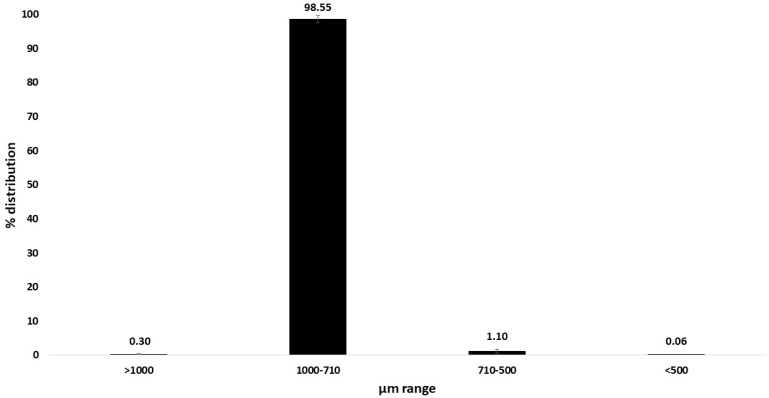
Particle size distribution of the microparticles obtained via ionotropic gelation.

The assessment of the particle size distribution is crucial because heterogeneity in this attribute may lead to problems such as non-reproducible results, dustiness, and variations in the bioactive release performances [26]. More than 98% of the MPs were in the range 710–1000 µm, indicating a very narrow particle size distribution of the product and the reliability of this technique for the production of MPs (Figure 3).

Alginate MPs showed good-fair flowability, according to the European Pharmacopoeia [25], since the mean angle of repose was 35.17 ± 3.82°. Therefore, in this case, these results indicated the appropriateness of the product for the intended final use.

High residual moisture can adversely affect the powder’s flowability and the release profile of the bioactive compound. Moreover, excessive moisture may promote microbiological growth; therefore, the residual water content in the final solid product should be kept low to minimize the risk of bacterial contamination.

**Figure 4:**
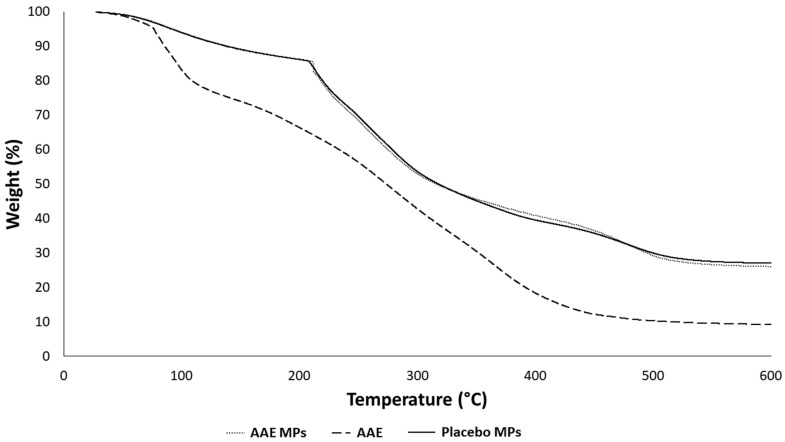
TGA profiles of AAE MPs (*A. absinthium* L. extract loaded microparticles), AAE (*A. absinthium* L. extract) and placebo MPs (microparticles).

The residual water content of alginate MPs was evaluated via thermogravimetric analysis and its low value (<9%) demonstrated the efficiency of the drying method. TGA profiles (Figure 4) indicated that the presence of absinthe extract in the MPs did not affect the thermal degradation profile of the MPs’ whole structure. The thermal profile of the loaded MPs showed the first thermal event at low temperature (up to 125 °C) due to water evaporation, then a remarkable signal between 200–215 °C, attributable to the degradation of alginate, and at higher temperatures, the degradation of all the other components occurred. There were no signals attributable specifically to absinthe.

**Figure 5:**
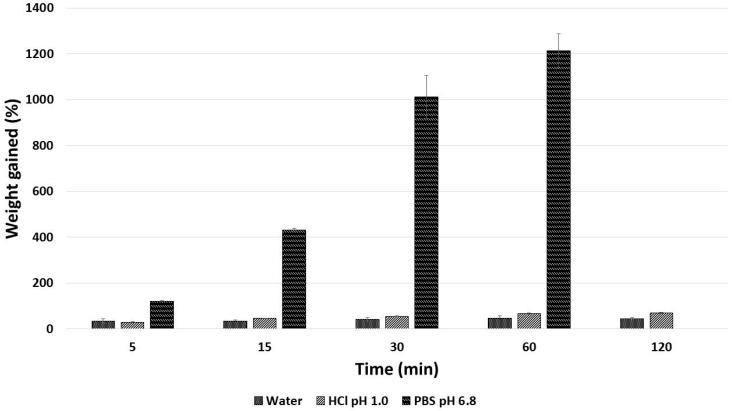
Swelling test results.

The swelling behaviour of dried alginate MPs was evaluated in three media: water, hydrochloric acid solution pH 1.0 and phosphate buffer solution 0.1 M pH 6.8 (Figure 5).

When MPs were in contact with PBS, they rapidly gained weight (120% after 5 min, 1200% after 1 h), increased in volume and in the end, within two hours, they were completely disaggregated in the fluid. The ion exchanges between the Na^+^ ions present in the PBS and the Ca^2+^ ions binding the carboxylic groups of alginate in the egg-box structure led to an increased electrostatic repulsion among negatively charged –COO^−^ groups with consequent chain relaxation and enhanced water penetration, up to the resulting disorganization of polymeric chains and disaggregation of the matrix structure of MPs. On the contrary, the MPs placed in water and HCl did not gain much weight (max 44% and 69%, respectively, after 2 h) nor disaggregate throughout the test. In an acidic medium, alginate formed acidic gels that could hinder fluid uptake, leading to a lower and slower weight gain than in PBS [27]. In water, the MPs showed an even minor tendency to uptake the fluid and swell, and this small gain in weight may be mainly attributed to the hydration of the hydrophilic group of alginate [28]. Nevertheless, these values related to the tendency to swell in HCl and water were slightly higher than those reported in the literature and this behaviour may be due to the presence of Plp 90G that could enhance water penetration within the alginate polymeric chains.

**Figure 6:**
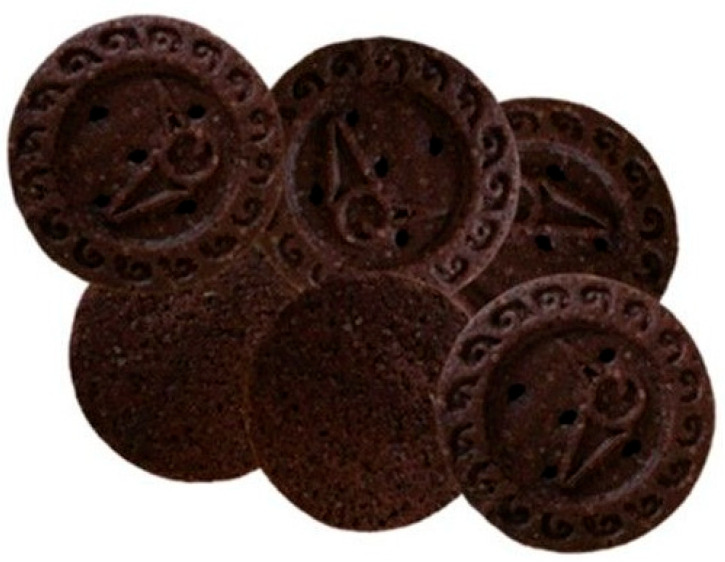
Biscuits containing AAE-loaded MPs.

Since ionotropic gelation proved to be the most promising technique for obtaining microparticulate systems applicable for the intended uses, it was selected to produce a larger batch capable of satisfying the small-scale production of biscuits. The required amount of MPs containing *A. absinthium* extract was successfully produced, delivered to Albertengo S.p.a., and used as an ingredient for the production of absinthe-enriched cocoa biscuits (Figure 6). Cocoa powder was used as an ingredient because it could help in masking the absinthe’s bitter taste, along with the microencapsulation approach.

### 2.4. Biscuits Characterization

#### 2.4.1. Proximate Composition

Proximate composition of biscuits produced is reported in the Supplementary Materials Table S1. Composition analysis resulted in slightly higher values of moisture, lipid and total dietary fiber content in cocoa biscuits enriched with the AAE MPs compared with cocoa biscuits, although no significant differences were observed. Protein content was significantly higher in the AAE MPs-containing biscuits, even though this difference is not so prominent in terms of absolute value.

#### 2.4.2. Total Phenols Content and Antioxidant Activity

The Folin–Ciocâlteu assay revealed comparable TPC between CBs and cocoa biscuits enriched with AAE MPs (MPBs), with values of 2.00 and 2.03 mg catechin equivalents (CE)/g, respectively, indicating that the incorporation of MPs did not significantly affect the overall phenolic concentration (Table S2). The overall phenolic composition of cocoa biscuits is attributed to the presence of flavan-3-ols, which are correlated with positive health effects [29]. In contrast, the DPPH assay showed a slight but statistically significant reduction in AA in MPBs (1.38 mg Trolox equivalents (TE)/g) compared with CBs (1.57 mg TE/g), suggesting a modest decrease in radical-scavenging capacity. This reduction may be attributable to interactions between phenolic compounds and the alginate matrix of the MPs. Alginate hydrogels can physically trap phenolics and promote hydrogen bonding or electrostatic interactions that limit phenolic accessibility or reactivity toward the DPPH radical, even when their total concentration remains unchanged. Such effects have been observed in alginate microbeads used to encapsulate phenolic antioxidants, where the polymer network influences the measured antioxidant activity by modulating release and reactivity in radical assays like DPPH [30].

#### 2.4.3. FAME Analysis

GC–FID profiling of fatty acid methyl esters (Table S3) was carried out to verify whether the incorporation of AAE MPs influenced the lipid fraction of the biscuits.

In both formulations, MUFAs predominated, largely driven by oleic acid (C18:1), which accounted for 65.55% in CBs and 66.53% in MPBs of the total fatty acids identified. Among PUFAs, linoleic acid (C18:2ω6cis) was the main component (12.07% and 12.27%, respectively), followed by ω3 PUFA α-linolenic acid (C18:3ω3) (2.40% in CBs and 2.43% in MPBs). Regarding SFAs, palmitic acid (C16:0) represented the major saturated species in CBs and MPBs (10.61% *vs* 10.81%), followed by stearic acid (C18:0) (3.48% *vs* 3.50%) and minor amounts of arachidic acid (C20:0) (0.51% *vs* 0.49%). When individual fatty acids were grouped, class sums were likewise very similar between formulations (CBs *vs* MPBs: SFA 14.60% *vs* 14.80%, MUFA 65.94% *vs* 66.92%, PUFA 14.85% *vs* 15.08%).

The predominance of C18:1, together with C16:0, among the major fatty acids reflects the main lipid source used in the biscuit formulation (90% extra-virgin olive oil and 10% hemp oil) and is consistent with the typical fatty acid composition of extra-virgin olive oil (oleic acid 55.0–83.0% and palmitic acid 7.5–20.0%), which varies with variety and fruit ripening stage.

Overall, the enrichment did not significantly affect the fatty acid composition: no statistically significant differences (*p* > 0.05) were observed between CBs and MPBs either for individual fatty acids or for the main fatty acid classes (SFA, MUFA, and PUFA). These results indicate that the lipid profile of the final products was essentially conserved after fortification and processing, consistent with the biscuit fat matrix remaining the primary determinant of fatty acid composition, with a negligible contribution of microparticles to total lipids.

From a nutritional perspective, the balance between ω6 and ω3 PUFAs was maintained: the ω6/ω3 ratio was 5.19 in CBs and 5.20 in MPBs, reinforcing the conclusion that enrichment did not induce a measurable shift in lipid class distribution [31].

#### 2.4.4. Quantification of Bitter Compounds Extracted from Biscuits

Absinthin and anabsinthin extracted from the MPs contained in the biscuits were quantified via HPLC-DAD and the results were respectively 356.8 ± 1.0 ng/g and 45.0 ± 1.5 ng/g.

Considering the consumption of 30 g of biscuits per day and that the average weight of one biscuit is 5.89 g, the total amount of the bitter compound absinthin ingested would be 10.510 µg per day (5 biscuits/day).

### 2.5. Consumer Acceptance

The biscuits with and without microencapsulated bitter compounds were used to conduct an acceptance test. They were submitted to consumers to evaluate their perception of the microencapsulated product when inserted into the biscuits. In Table 1, the results of the consumer test are reported as the sum of ranks calculated for each type.

Among all the sensory parameters evaluated by the consumers, the only significant differences (*p* < 0.05) between the two formulations were related to taste and flavour, meaning that the addition of absinthe did not influence the tasters’ judgment of the biscuit’s appearance, odour and texture, which resulted in equal rankings. In terms of taste, the control biscuits (CBs) were rated higher than those containing the encapsulated extract of *A. absinthium*. The mean score of the judges was 5.36 ± 1.27 in the case of CBs, while that for MPBs was 4.68 ± 1.35. Therefore, the addition of the bitter compound has a negative effect on the taste of the product, but only a slight one. Consumers perceived the presence of a different bitter taste, which they did not associate with cocoa due to its more vegetal notes. In terms of flavour, again, CBs was the most appreciated sample with a mean of 5.74 ± 1.79, followed by the MPBs with a mean of 4.44 ± 2.31. The negative judgment was more related to the type of oil used in the recipe. It was a mixture of extra virgin olive oil (90%) and hemp oil (10%) and the standard Italian consumer is not used to the strong vegetable notes of the hemp oil.

The overall liking was related to the individual sensory parameters analysed, with the CBs being preferred to MPBs, but these differences were not statistically significant and both samples received a neutral or positive judgment.

Therefore, the formulation created could be a good strategy to use for the introduction of *A. absinthium* extract as a functional ingredient, but a different and more well-known oil should be evaluated to have greater acceptance by consumers.

## 3. Conclusions

*Artemisia absinthium* L. ethanolic extract was successfully microencapsulated and ionotropic gelation turned out to be a reliable method for the production of homogeneous and intact alginate-based MPs. The MPs containing absinthe extract were successfully inserted into biscuits and these products were, in general, non-negative and not worse than the control biscuits. Both types of biscuits produced had an excellent ω6/ω3 ratio between the unsaturated fatty acids, and they may be included in healthy diets. Finally, biscuits containing bitter compounds may be used to verify the decrease in the sense of hunger and if positive results are obtained, they may be incorporated into some diets to reduce caloric intake. Additional data concerning hunger-related hormones are required to confirm this hypothesis, and these experiments will be the subject of future research work.

## 4. Materials and Methods

### 4.1. Materials

*Artemisia absinthium* L. was provided by the Botanical Garden of Guardabosone (Italy). A voucher specimen (GBAb-22) of the *cis*-epoxyocimene chemotype of *A. absinthium* is stored in the Laboratory of Phytochemical Compounds, Department of Pharmaceutical Sciences, University of Eastern Piedmont, Novara, Italy. Silica gel 60 (70–230 mesh) used for gravity column chromatography was purchased from Macherey-Nagel (Düren, Germany).

Chemical reagents and solvents were from Aldrich (Darmstadt, Germany) and were used without any further purification unless stated otherwise. ^1^H NMR 400 MHz spectra were measured on Bruker 400 spectrometers (Bruker, Billerica, MA, USA). Chemical shifts were referenced to the residual solvent signal (CDCl_3_: δ_H_ = 7.26). Phospholipon^®^ 90G (phosphatidylcholine) and Phospholipon^®^ 90H (phosphatidylcholine hydrogenated) were kindly donated by Lipoid GmbH (Ludwigshafen, Germany). Ethylcellulose was purchased from Sigma Aldrich (St. Louis, MO, USA), sodium carboxymethyl cellulose was purchased from FMC BioPolymer (San Colombano al Lambro, MI, Italy), hydroxypropyl cellulose (Lot A015194701) was purchased from Acros organics (Bridgewater, NJ, USA), Methocel^®^ (hydroxypropyl methylcellulose HPMC) was purchased from Colorcon (Kent, UK). Sodium alginate (mannuronic (M) and guluronic (G) residues ratio 1.8–2.2, viscosity 1% solution 500–600 mPa·s) was purchased from Farmalabor (Assago, MI, Italy), and Tween 80 was purchased from Sigma Aldrich (St. Louis, MO, USA). Tripalmitin was kindly donated by IOI Chemical (Johor, Malaysia), palmitic acid was purchased from Fluka, and beeswax was kindly donated by Farmalabor (Assago, MI, Italy).

All other reagents were of analytical grade and used as received.

### 4.2. Experimental Methods

#### 4.2.1. Ethanolic Extraction of *Artemisia absinthium* L.

Leaves and flowers (1500 g) of *A. absinthium* L. were all extracted with ethanol (1:10, *w*/*v*, 2 × 12 h) in a vertical stainless-steel percolator at room temperature to obtain the *A. absinthium* L. extract (AAE), after solvent evaporation at reduced pressure. AAE was then characterised for the main sesquiterpene lactones using ^1^H NMR.

#### 4.2.2. Characterization of AAE

5 g of AAE were fractionated by low-pressure chromatography (LPC) on silica gel (100 g, petroleum ether (PE)/ethyl acetate (EtOAc) gradient from 90:10 to 20:80) to identify and quantify the main sesquiterpene lactones. Purification was monitored by TLC on Merck 60 F254 (0.25 mm) plates (Sigma Aldrich, St. Louis, MO, USA), using a 30:70 PE/EtOAc mixture as the eluent, and visualized via staining with 5% H_2_SO_4_ in EtOH and heating. All the molecules were identified via ^1^H NMR as previously described in the literature [32].

##### Total Phenolic Content (TPC) and Antioxidant Activity (AA) of the Extract

The TPC and AA of the ethanolic starting extract were determined by Folin–Ciocâlteu assay and by the 2,2-azino-bis-3-ethylbenzothiazoline-6-sulphonic acid (ABTS) radical cation decolorization assay, respectively, following the protocols described in the literature with some modifications [33,34].

Briefly, for the TPC, 50 µL of Folin–Ciocâlteu reagent was added. Then, 10 µL of a diluted sample solution and 175 µL of a sodium carbonate solution (5% *w*/*v*) were added. Finally, deionized water was added to reach a final volume of 1450 µL. The cuvette was stored in the dark for 60 min, then the absorbance was determined at 760 nm by a UV–Vis spectrophotometer (UV-1900 model, Shimadzu, Beijing, China). Results were expressed as mg of catechin equivalents (CE) per mL of extract using a calibration curve with catechin (y = 0.0171x + 0.0033, R^2^ = 0.993).

ABTS^+^ was prepared by reacting a 7 mM ABTS solution with 2.45 mM potassium persulphate, followed by incubation for 16 h in the dark at room temperature. Before initiating the assay, the ABTS solution was diluted with ethanol to an absorbance of 0.700 ± 0.020 at 734 nm. 10 µL of each sample was mixed with 1 mL of ABTS^+^ solution and it was allowed to react for 6 min. Absorbance was read at 734 nm and results were expressed as mg of Trolox equivalents (TE) per mL through a calibration curve (y = 17.793x − 2.1504, R^2^ = 0.997)

#### 4.2.3. Preliminary Formulation Studies

Firstly, since the dry extract was challenging to handle, it was completely solubilized in an adequate amount of solvent, acetone or ethanol, depending on the different production technique that was intended to be used.

A preliminary study was conducted to investigate the workability of different production technologies, specifically spray drying and ionotropic gelation, to obtain microparticulate systems.

##### Spray Drying

Regarding preliminary evaluation for the feasibility of obtaining AEE-loaded microparticulate systems by spray drying, different formulations containing cellulose derivatives (carboxymethylcellulose CMC, ethylcellulose EC, hydroxypropyl cellulose HPC, hydroxypropyl methylcellulose HPMC) solubilized in two solvents (acetone and ethanol) at various concentrations were tested and the best ones in terms of viscosity and solid content were selected and submitted to spray drying treatment. Process recovery was expressed as a percentage and it was calculated according to the following Equation (1):

(1) Recovery %=Wc/Ws∗100

where Wc is the weight of the powder obtained and Ws is the sum of the weights of the components of the formulation, except for the solvent.

##### Ionotropic Gelation

As far as ionotropic gelation was concerned, different formulations, including two stabilizers (phosphatidylcholine and Tween 80), were investigated and the formulation with the best stability and viscosity was selected as suitable for this microencapsulation technology.

#### 4.2.4. Preparation of the Microparticles

##### Spray Drying

Eight formulations, differing in AAE loading, solvent used, and process conditions, were submitted to spray drying (Table 2) using a Büchi Mini Spray Dryer B-290 (Büchi Labortechnik AG, Flawil, Switzerland) and the exhausted solvent was sorted directly under an active chemical fume hood. AAE was solubilized in acetone (formulations 1 to 4) or ethanol (formulations 5 to 8). Then, ethylcellulose was added at exact ratios to AAE (1:1 and 1:2). The resulting formulations were stirred under magnetic stirring to obtain homogeneous liquid preparations, which were then spray-dried. Process conditions varied across formulations (Table 3). The obtained MPs were then characterised for morphology, dimensions, and flowability, and the process recovery was calculated.

##### Ionotropic Gelation

Ionotropic gelation, exploiting the crosslinking reaction between the carboxylic residues of sodium alginate and divalent calcium ions, was carried out.

Briefly, sodium alginate and phosphatidylcholine (Phospholipon^®^ 90G) were dissolved in water. Then, the dry extract of *A. absinthium,* completely solubilized in ethanol, was added to the polymeric solution under magnetic stirring to obtain a homogeneous dispersion. Microspheres were formed by dripping the dispersion through a two-fluid nozzle equipped with a 700 µm tip into a 100 mM CaCl_2_ solution, followed by curing (15 min), filtration and rinsing. These conditions were selected according to our previous experiments. Wet MPs were characterized for morphology and dimensions. Dried MPs were obtained by fluid bed drying at 25 °C (classical air distributor, air flow = 35 m^3^/h) or by static drying at room temperature on a Petri dish and the differences between the two drying methods on the dried MPs’ morphological aspect were investigated via SEM. Fluid bed-dried MPs were completely characterized and the process recovery was calculated.

#### 4.2.5. Characterization of the MPs

##### Morphology and Particle Size

The morphology and size of MPs obtained via spray drying, and of wet and dried MPs obtained by ionotropic gelation, were investigated by stereomicroscopy (Stereomicroscope Leica S9, Milan, Italy) and image analysis was performed using ImageJ software 1.501 (National Institutes of Health, Bethesda, MD, USA). Dried MPs obtained by ionotropic gelation were also characterized for morphology by scanning electron microscopy analyzing the samples at 5–6 kV after coating with a 4 nm Au layer (SEM, Phenom XL, Thermo Fischer Scientific, Waltham, MA, USA).

The particle size distribution of the dried alginate MPs was assessed by sieving. Sieves with different decreasing mesh (35, 25, 18, Giuliani Tecnologie S.n.c., Torino, Italy) were piled up and the MPs were placed on top of the stack. Then, the sample was subjected to mechanical vibration and at the end of the test, the MPs fractionated for their dimensional range were weighed and the size distribution was calculated according to the following Equation (2):

(2) $$Fraction\;\%=\frac{Wf}{Wt}*100$$

where *Wf* is the weight of the fraction of MPs included in the selected dimensional range and *Wt* is the total weight of the MPs produced.

The results are the average of three determinations.

##### Flowability

The flowability of the powders produced by spray drying and of the dried microspheres obtained via ionotropic gelation was evaluated by determining the dynamic angle of repose, as specified in the European Pharmacopoeia [25]. Briefly, an amount of 100 mg of the sample was placed into a cylindrical container (3.0 cm in diameter and 1.5 cm in height) and assembled onto a rotating rod stirring parallel to the horizontal axis. The instrument was turned on and the rotation speed was set at 50 rpm. After 20 s, a camera placed in front of the cylinder started to record a video, from which different pictures (at least 12 for each sample) were taken to evaluate the dynamic angle of repose by using the ImageJ software “angle tool”.

##### Residual Moisture and Thermal Behaviour

The residual moisture and thermal behaviour of alginate dried MPs were determined by thermogravimetric analysis (TGA, Perkin Elmer, Pyris 4000, Shelton, CT, USA). A small amount of sample (about 20 mg) was placed onto the balance weighing pan and a ramp from 25 to 700 °C, with a scan rate of 10 °C/min was set in a N_2_ atmosphere. Residual moisture was calculated just taking into consideration the weight loss in the interval 25–125 °C.

##### Swelling Test

The swelling behaviour of dried alginate MPs in three different media was evaluated to understand how they could behave. An exact weighed amount of dried alginate MPs was put in contact with 5 mL of water (H_2_O), hydrochloric acid (HCl) pH 1.0, or phosphate buffer solution (PBS) pH 6.8, at room temperature. At predetermined time intervals (5, 15, 30, 60, 120, and 240 min), MPs were collected, gently dabbed with blotting paper and re-weighed to assess the eventual weight increase due to the fluid uptake. The following Equation (3) was used to calculate the percentage of swelling:

(3) $$Sw=\frac{Wt-W0}{W0}*100$$

where Wt is the sample weight after having been in contact with the fluid and W0 is its initial weight.

Each test was conducted in triplicate and the results are the average of three determinations.

#### 4.2.6. Production of the Biscuits

Two types of biscuits were produced in the pilot plant of the Albertengo S.p.A. company (Torre San Giorgio, CN, Italy): a biscuit control batch without MPs (control biscuits, namely “CBs”) and a batch of biscuits loaded with 0.1 kg of the most performing MPs, i.e., AAE alginate fluid bed dried MPs (MP-containing biscuits, namely “MPBs”). The recipes consisted of wheat flour “type 00” 3.600 kg, white granulated sugar 2.400 kg, vegetable oil mixture 1.750 kg (90% extra virgin olive oil, 10% hemp oil), Primafibra^®^ fiber 1.200 kg, water 0.750 kg, bitter cocoa powder 0.450 kg, whole egg mix 0.400 kg, pure vanillin flavoring 0.024 kg, fine marine salt 0.017 kg, and ammonium bicarbonate 0.012 kg, resulting in 10.603 kg and 10.703 kg of raw total, for CBs and MPBs recipe, respectively. Finally, 7.000 kg of total baked product were produced for each recipe. The process consisted of mixing all the ingredients with a planetary mixer (RAM K60I, Ram S.r.l., Schio, VI, Italy) for 5 min; then the dough was shaped using a rotary moulder machine, and the obtained biscuits were cooked in a ventilated electric oven (tunnel oven Polin, Ing. Polin E C. S.p.A., Verona, Italy) for 8 min at 160 °C.

#### 4.2.7. Biscuits Characterisation

##### Proximate Composition

Biscuits were characterised as regards moisture, protein, lipid and total dietary fiber content.

Moisture content was determined utilizing a thermogravimetric balance (Sartorius MC30, Goettingen, Germany). Protein content was evaluated following the Kjeldahl method, using Kjeltec system I (Foss Tecator AB, Höganäs, Sweden). Lipid content was assessed through a continuous Soxhlet extraction using a Büchi Extraction System B-811 (BÜCHI Labortechnik AG, Flawil, Switzerland) in dichloromethane. Total dietary fiber (TDF) was determined following the method AOAC 991.43 [35], and utilizing the “total dietary fiber assay” kit (Megazyme, Bray, Ireland). All the analyses were performed in triplicate.

##### Extraction of the Phenolic Fraction from the Biscuits

The phenolic fraction was extracted from previously ground and defatted biscuits. The phenolic fraction was obtained by treating the samples with an ethanolic solution 50% *v/v* under stirring followed by an ultrasonic bath (Branson 1510, Branson Ultrasonics, Danbury, CT, USA) for 2 min. Samples were then centrifugated for 3 min at 14,000 rpm (Eppendorf Centrifuge 5417R, Eppendorf, Milano, Italy) and the supernatant was collected. The procedure was repeated three times.

##### Total Phenolic Content and Antioxidant Activity of the Biscuits

The TPC and AA of the defatted biscuits were evaluated. TPC was determined by Folin–Ciocâlteu assay following the procedure previously described, while AA was determined through the DPPH^•^ assay as reported by Disca et al. [33]. Briefly, 700 µL of opportunely diluted sample or methanol (control) was added to the same volume of a 100 µmol/L DPPH^•^ methanolic solution. The solution was left in the dark at room temperature for 20 min, and the absorbance was read at 515 nm. Extracts were subjected to triplicate assays, and results were expressed as milligrams of Trolox equivalents (TE) per gram of biscuits through a calibration curve.

##### Fatty Acids Methyl Esters (FAME) Analysis

Fatty acid methyl esters were prepared from triacylglycerols through a transesterification procedure modified from Locatelli et al. [36]. In brief, 0.20 g of the lipid extract was reacted with 200 μL of 0.5 mol/L sodium methoxide. The mixture was incubated in a sealed vial at 80 °C with continuous agitation at 350 rpm for 10 min using a Thermomixer (Eppendorf, Milan, Italy). Upon completion of the reaction, distilled water (250 μL) was added, followed by diethyl ether (500 μL) to promote phase separation. The vial was gently mixed to facilitate the extraction of the methyl esters. An aliquot (50 μL) of the organic phase was subsequently diluted with 950 μL of dichloromethane before analysis. Chromatographic separation and detection of FAMEs were performed using a Shimadzu GC-17A gas chromatograph (Shimadzu Italia, Milan, Italy) fitted with a split/splitless injector, a flame ionization detector, and a DB-23 capillary column (30 m × 0.25 mm i.d., 0.25 μm film thickness; J&W Scientific, Folsom, CA, USA). Both the injector and detector temperatures were maintained at 250 °C. Hydrogen served as the carrier gas at a constant flow rate of 1.5 mL/min, with a split ratio of 50:1. Individual fatty acids were identified by comparison of retention times with those obtained from a standard FAME mixture (Supelco 37 Component FAME Mix, Sigma-Aldrich, Steinheim, Germany). Quantitative results were expressed as relative percentages, calculated from the total peak area.

##### Extraction and Analysis of Bitter Compounds

Bitter compounds were extracted via liquid-liquid extraction (LLE) performed on ground biscuit samples. LLE was carried out using chloroform as the extraction solvent, after disaggregation of the MPs in phosphate buffer 0.2 M pH 6.8.

HPLC-DAD analysis was performed to identify the main *A. absinthium* bitter compounds, according to the method reported by Aberham et al. [32] with some modifications, using a Shimadzu LC-20A Prominence (Shimadzu, Canby, OR, USA), equipped with a diode array detector (DAD detector SPD-M20A, Shimadzu, Canby, OR, USA). A column Phenomenex Luna C-18(2) (150 × 2 mm, pores diameter 100 Å) (Phenomenex, Castel Maggiore, BO, Italy) was used. Mobile phase A consisted of water containing 0.1% (*v*/*v*) formic acid, while mobile phase B consisted of acetonitrile containing 0.1% (*v*/*v*) formic acid. The column temperature was maintained at 35 °C, and analyses were performed at a flow rate of 0.5 mL/min with a total run time of 45 min. The gradient elution program was as follows: 30% B at 0 min; linear increase from 30% to 50% B over 10 min; linear increase from 50% to 70% B over the next 10 min; linear increase from 70% to 98% B over 3 min; isocratic hold at 98% B for 7 min; return to 30% B over 3 min, followed by a 12-min re-equilibration at 30% B. Detection was carried out at 205 nm. Absinthin and anabsinthin were used as standards and calibration curves were prepared. Absinthin calibration curve: y = 4293.3x − 844.77, R^2^ = 0.996. Anabsinthin calibration curve: y = 44,404x − 5309.2, R^2^ = 0.998. Results are reported as nanograms of bitter compound per gram of dried product (ng/g) and they are the average of three determinations.

#### 4.2.8. Consumer Evaluation

The study was approved by the ethical committee of AOU della Carità on (CE 054/2022, amended code E00003/2022), and it is also registered on clinicaltrial.gov (NCT05528874). The protocol is conducted in accordance with the principles of the Declaration of Helsinki and EU Clinical Good Practice Standards. Before the beginning of any procedures, recruited healthy subjects signed a written informed consent for the study participation and a second one for biobanking at the UPO Biobank. Participants had the right to give or withdraw consent at any time.

A consumer acceptance evaluation was performed by a panel of 50 untrained volunteer tasters (30% male, 70% female, 20–65 years old), to whom biscuits were given in random order. Tasters were asked to evaluate the following parameters: appearance, odor, taste, flavor, texture and overall liking using a 9-point hedonic scale (1 = extremely dislike, 5 = neither like nor dislike, 9 = extremely like). The test was carried out in an air-conditioned room (21 °C) equipped with a white light. Water was provided for mouth rinsing to neutralize the taste between sample testing.

The Kruskal–Wallis H-test (95% confidence level) with a multiple comparison test was applied for the consumer acceptance evaluation using SPSS Statistics 29 software (IBM-SPSS Inc., Chicago, IL, USA).

## Figures and Tables

**Table 1:** Results of the Kruskal–Wallis test (reported as the sum of ranks) of consumer acceptance evaluation and results of the multiple comparison test (CBs = control biscuits, MPBs = biscuits containing microparticles). Data followed by different letters are significantly different at *p* < 0.05.

	Appearance	Odor	Taste	Flavor	Texture	Overall Liking
CBs	2269 ^a^	2588 ^a^	2878 ^b^	2927 ^b^	2422 ^a^	2757 ^a^
MPBs	2781 ^a^	2462 ^a^	2172 ^a^	2124 ^a^	2628 ^a^	2293 ^a^

**Table 2:** Composition of formulations submitted to spray drying (AAE = *A. absinthium* L. extract; EC = ethylcellulose).

Formulation	1	2	3	4	5	6	7	8
Solvent (%)	86.7	79.7	83.9	79.7	92.9	90.8	91.3	88.8
AAE (%)	4.4	10.2	5.4	10.2	2.3	4.6	2.9	5.6
EC (%)	8.9	10.2	10.7	10.2	4.7	4.6	5.8	5.6

**Table 3:** Spray drying process parameters. P nozzle = pressure of atomization, T = temperature.

Formulation	1	2	3	4	5	6	7	8
Feed rate (mL/min)	3.8	4.0	3.7	3.7	3.7	3.8	4.1	3.9
P nozzle	30	30	45	45	30	30	45	45
T inlet (°C)	65	65	65	65	90	90	95	95
T outlet (°C)	50	46	46	48	61	60	63	64
Tip hole (mm)	0.7	0.7	2.0	2.0	0.7	0.7	2.0	2.0
Load (%)	33.3	50.0	33.3	50.0	33.3	50.0	33.3	50.0

## Data Availability

The original contributions presented in the study are included in the article; further inquiries can be directed to the corresponding author.

## Supplementary Materials

The following supporting information can be downloaded at: https://www.mdpi.com/article/10.3390/gels12010043/s1, Table S1. Protein, lipid, and total dietary fiber (TDF) contents are expressed as percentages on a dry weight basis, whereas moisture is expressed on a wet basis. Values are reported as mean ± standard deviation (n = 3). CBs: cocoa biscuits; MPBs: cocoa biscuits + AAE MPs. Table S2. Total Phenolic Content (TPC) expressed as mg/g of catechin equivalents. Antioxidant activity (AA) expressed as mg/g of Trolox equivalents. (n = 3). Whereas present, different letters in the same row indicate samples significantly different. CBs: cocoa biscuits; MPBs: cocoa biscuits + AAE MPs. Table S3. Fatty acid composition determined by GC–FID and expressed as relative percentage (%) of total fatty acid methyl esters (FAMEs). Data are reported as mean ± standard deviation (n = 3). SFA: saturated fatty acids; MUFA: monounsaturated fatty acids; PUFA: polyunsaturated fatty acids; CBs: cocoa biscuits; MPBs: cocoa biscuits + AAE MPs.
