# Supplementary material for: Alginate-Based Beads Containing Artemisia absinthium L. Extract as Innovative Ingredients for Baked Products
Source: Gels. 2026 Jan 1;12(1):43. doi: 10.3390/gels12010043 (PMC12840771; doi:10.3390/gels12010043)
Supplement: Supplementary file 1 [file gels-12-00043-s001.zip › gels-4052203-supplementary.pdf]

Alginate-Based Beads Containing *Artemisia absinthium* L. Extract as Innovative Ingredients for Baked Products

**Alessandro Candiani [1], Giada Diana [1], Vincenzo Disca [1], Yassine Jaouhari [1], Margherita Stampini [1], Stefano Salamone [1], Federica Pollastro [1], Jessica Baima [2], Flavia Prodam [2], Sabrina Tini [2], Marta Bertolino [3],
Lorella Giovannelli [1], Lorena Segale [1,*], Jean Daniel Coïsson [1] and Marco Arlorio [1]**

[1] Department of Pharmaceutical Sciences, University of Piemonte Orientale, Largo Donegani 2, 28100 Novara, NO, Italy; alessandro.candiani@uniupo.it (A.C.); giada.diana@uniupo.it (G.D.); vincenzo.disca@uniupo.it (V.D.); yassine.jaouhari@uniupo.it (Y.J.); margherita.stampini@uniupo.it (M.S.); stafano.salamone@uniupo.it (S.S.); federica.pollastro@uniupo.it (F.P.); lorella.giovannelli@uniupo.it (L.G.); jeandaniel.coisson@uniupo.it (J.D.C.); marco.arlorio@uniupo.it (M.A.)

[2] Department of Health Sciences, University of Piemonte Orientale, Via Solaroli 17, 28100 Novara, NO, Italy; jessica.baima@uniupo.it (J.B.); flavia.prodam@uniupo.it (F.P.); sabrina.tini@uniupo.it (S.T.)

[3] Department of Agricultural, Forest and Food Sciences, University of Torino, Largo Paolo Braccini 2, 10095 Grugliasco, TO, Italy; marta.bertolino@unito.it

* Correspondence: lorena.segale@uniupo.it; Tel.: +39-0321-375868

Table S1. Protein, lipid, and total dietary fiber (TDF) contents are expressed as percentages on a dry weight basis, whereas moisture is expressed on a wet basis. Values are reported as mean ± standard deviation (n = 3). CBs: cocoa biscuits; MPBs: cocoa biscuits + AAE MPs.

(%)	**CBs**	**MPBs**
Moisture	3.63 ± 0.23	3.74 ± 0.29
Protein	15.81 ± 0.05	15.98 ± 0.02
Lipid	19.97 ± 0.55	20.61 ± 1.16
TDF	8.09 ± 0.50	8.62 ± 0.12

Table S2. Total Phenolic Content (TPC) expressed as mg/g of catechin equivalents. Antioxidant activity (AA) expressed as mg/g of Trolox equivalents. (n=3). Whereas present, different letters in the same row indicate samples significantly different. CBs: cocoa biscuits; MPBs: cocoa biscuits + AAE MPs.

	**CBs**	**MPBs**
TPC (mg CE/g)	2.00 ± 0.06	2.03 ± 0.01
AA (mg TE/g)	1.57 ± 0.02[b]	1.38 ± 0.02[a]

Table S3. Fatty acid composition determined by GC–FID and expressed as relative percentage (%) of total fatty acid methyl esters (FAMEs). Data are reported as mean ± standard deviation (n = 3). SFA: saturated fatty acids; MUFA: monounsaturated fatty acids; PUFA: polyunsaturated fatty acids; CBs: cocoa biscuits; MPBs: cocoa biscuits + AAE MPs.

Fatty acid	CBs	MPBs
	**SFA (%)**	
C16:0	10.61 ± 0.04	10.81 ± 0.05
C18:0	3.48 ± 0.03	3.50 ± 0.03
C20:0	0.51 ± 0.01	0.49 ± 0.02
	**MUFA (%)**	
C18:1	65.55 ± 1.19	66.53 ± 0.46
C20:1	0.39 ± 0.04	0.39 ± 0.02
	**PUFA (%)**	
C18:2ω6cis	12.07 ± 0.20	12.27 ± 0.08
C18:3ω6	0.38 ± 0.01	0.38 ± 0.01
C18:3ω3	2.40 ± 0.06	2.43 ± 0.02
ω6/ω3	5.19	5.20
